# New Insights into the Hendra Virus Attachment and Entry Process from Structures of the Virus G Glycoprotein and Its Complex with Ephrin-B2

**DOI:** 10.1371/journal.pone.0048742

**Published:** 2012-11-05

**Authors:** Kai Xu, Yee-Peng Chan, Kanagalaghatta R. Rajashankar, Dimple Khetawat, Lianying Yan, Momchil V. Kolev, Christopher C. Broder, Dimitar B. Nikolov

**Affiliations:** 1 Structural Biology Program, Memorial Sloan-Kettering Cancer Center, New York, New York, United States of America; 2 Department of Microbiology and Immunology, Uniformed Services University, Bethesda, Maryland, United States of America; 3 The Northeastern Collaborative Access Team, Advanced Photon Source, Argonne National Laboratory, Argonne, Illinois, United States of America; Johns Hopkins University - Bloomberg School of Public Health, United States of America

## Abstract

Hendra virus and Nipah virus, comprising the genus *Henipavirus*, are recently emerged, highly pathogenic and often lethal zoonotic agents against which there are no approved therapeutics. Two surface glycoproteins, the attachment (G) and fusion (F), mediate host cell entry. The crystal structures of the Hendra G glycoprotein alone and in complex with the ephrin-B2 receptor reveal that henipavirus uses Tryptophan 122 on ephrin-B2/B3 as a “latch” to facilitate the G-receptor association. Structural-based mutagenesis of residues in the Hendra G glycoprotein at the receptor binding interface document their importance for viral attachments and entry, and suggest that the stability of the Hendra-G-ephrin attachment complex does not strongly correlate with the efficiency of viral entry. In addition, our data indicates that conformational rearrangements of the G glycoprotein head domain upon receptor binding may be the trigger leading to the activation of the viral F fusion glycoprotein during virus infection.

## Introduction

The henipaviruses are recently emerged highly pathogenic paramyxovirus zoonoses and include Hendra virus (HeV) and several distinct isolates of Nipah virus (NiV) [1]. HeV was first isolated in 1994 from specimens obtained during an outbreak of respiratory and neurologic disease in horses and humans in Hendra, a suburb of Brisbane, Australia[2]. To date, there have now been 39 recorded outbreaks of HeV infections, 25 of these in just the past two years the most recent in September of 2012 [3], all causing lethal respiratory disease and encephalitis in horses. Five of these events have also involved a total of seven human cases four of which were fatal [4]–[7]. Infections of NiV have also repeatedly occurred involving hundreds of human cases since its emergence in 1998 in a large outbreak of disease in humans and pigs, and there have been at least thirteen recognized occurrences in Bangladesh and India since 2001 the most recent in January of 2012[2]. The natural reservoir hosts of both HeV and NiV are fruit bats, predominantly several species of Pteropid bats (family *Pteropodidae*) [4]. The henipaviruses also possess a unique and very broad species tropism documented by both natural and experimental infections and in addition to bats, humans, horses and pigs, they can infect and cause disease in guinea pigs, hamsters, cats, dogs (reviewed in [4]), ferrets [8] and nonhuman primates [9], [10]. Because of their highly pathogenic characteristics and lack of any approved therapeutic approaches, the henipaviruses are classified as select agents and biosafety level 4 (BSL-4) pathogens [11].

For many enveloped viruses, entry is mediated by viral fusion glycoproteins that contain two distinct activities: receptor attachment and membrane fusion. The fusion activity is triggered either by receptor binding or exposure to an acidic environment following endocytosis [12]. In the paramyxovirus family, the attachment and membrane fusion activities are performed by two separate envelope glycoproteins [13]. The henipaviruses possess an attachment (G) and a fusion (F) glycoprotein which together work in concert to mediate the virus attachment and entry process, but the precise triggering mechanism of paramyxovirus fusion has yet to be defined in detail [14], [15]. The henipavirus G glycoproteins have a type-II transmembrane topology, containing a short N-terminal cytoplasmic tail and a long C-terminal extracellular globular head. These two domains are connected by transmembrane and extracellular stem regions, and membrane anchored G forms disulfide-linked dimers which associate in pairs as a tetrameric oligomer [16]. Distinct from most other members within the subfamily *Paramyxovirinae*, the henipavirus attachment glycoprotein does not hemagglutinate and neither binds to sialic acid, nor retains neuraminidase activity, and instead binds cell surface protein receptors [1]. Recently, ephrin-B2 and ephrin-B3 were identified as the functional receptors for both HeV and NiV [17]–[20]. The F glycoprotein is a type-I transmembrane protein, which is initially synthesized as a precursor F_0_ which form trimeric oligomers that are then proteolytically processed into the disulphide-linked subunits F_1_ and F_2_ [21]. The direct association between paramyxovirus attachment glycoproteins with their respective F glycoprotein has also been reported, and important elements for this feature have been mapped to several sites in both the stem regions and the globular head domains among several virus species [22]–[31].

The pre-fusion trimeric F glycoprotein is proposed to be in a “metastable” conformation and associated with its oligomeric attachment glycoprotein partner. A current, and widely accepted, model of paramyxovirus fusion suggests that upon receptor binding, the F glycoprotein is activated, presumably involving direct contacts between the attachment and fusion glycoproteins, and inserts its fusion peptide into the host cell membrane. The activation process facilitates a series of conformational changes in F and the glycoprotein transitions into its post-fusion, six-helix-bundle conformation concomitant with the merging of the viral membrane envelope and the host cell plasma membrane [15]. However, all of the details of the entire receptor binding and fusion activation process have yet to be defined, and the structural characterization of the F and G glycoproteins across the various stages of these processes is essential for detailing this critical step in the virus life-cycle. The structures of HeV-G alone and the HeV-G/ephrin-B2 complex were recently published [32], [33], however, many important details were not revealed possibly due to the low resolution (3.3 Å for the complex). The high-resolution structures described here, including both HeV-G alone (2.2 Å) and in complex with ephrin-B2 (2.7 Å), combined with structure-based mutagenesis, reveal new insights into the molecular mechanisms governing the initial steps of henipavirus entry into host cells and into the paramyxovirus entry mechanism in general.

## Results and Discussion

### Structure of unbound HeV-G

**Figure 1:**
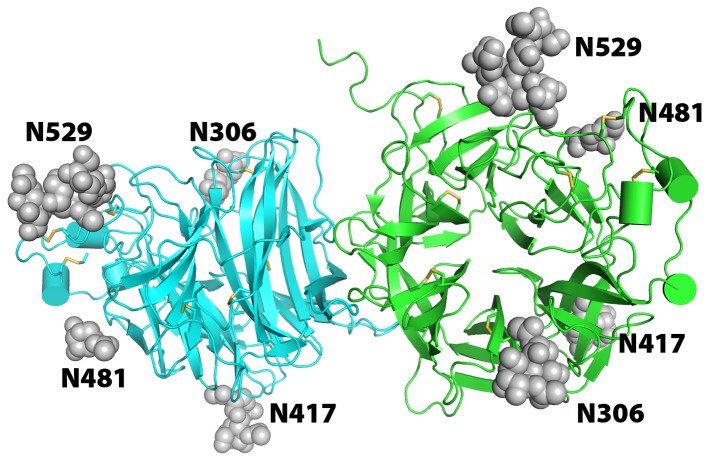
Structure of the HeV-G dimer. The secondary structure elements of the two molecules are colored in cyan and green. The axes of the two six-blade β-propellers are approximately perpendicular to each other. Disulfide bonds are illustrated as yellow sticks. Asparagine-linked carbohydrate modifications (glycosylations) are illustrated as grey spheres.

The HeV-G globular head domain (174–602) was expressed using the baculovirus/insect-cell system as described in the *Materials and Methods*. The structure was determined at 2.2 Å resolution (**Table S1**) using molecular replacement with NiV-G (PDB ID 3D11) as a search model. Similar to other paramyxovirus attachment proteins, the HeV-G's head domain folds as a six-blade (B1–B6) β-propeller (**Figure 1**). Each blade contains four anti-parallel beta-strands (S1–S4), except B6 (S1–S5), which is composed of the three C-terminal strands of the protein and its two most N-terminal strands, thus forming a “velcro”-type closure. The blades are connected through extended loops between S4 of one module and S1 of the next. There are three α-helices located inside B2 and B3, as well as between B6 and B1. The N and C termini of the HeV-G head domain are connected through a disulphide linkage (C189–C601). The structure is further stabilized by six additional disulphide bonds (C216–C240, C282–C295, C382–C395, C387–C499, C493–C503 and C565–C574), as well as by a number of hydrogen bonds and van der Waals interactions. The central cavity of the β-propeller is funnel-shaped with the bottom face covered by the N-terminal strand and loop. The upper face is open and available for receptor binding. We observed carbohydrate moieties at all five predicted N-linked glycosylation sites (N306, N378, N417, N481 and N529), but N378 was not modeled due to the weak electron density. The site occupancy of the predicted N-linked glycosylation sites and a detailed glycan composition analysis of recombinant soluble HeV G glycoprotein (sG) was discussed in [34].

While gel-filtration and analytical ultracentrifugation indicate that the HeV-G head domain is monomeric in solution, it packs as a dimer within one asymmetric unit in the crystals (**Figure 1**), with the axes of the two propellers forming an angle of approximately 90°. The dimeric interface is located in the B6S2–S3/B1H1-S1 surface region (**Figure 1**). Interestingly, similar packing arrangements were reported in other crystal structures of attachment proteins of the paramyxovirus family, such as HeV, NDV, PIV3, MeV and SV5 [33], [35]–[40]. The paramyxovirus attachment proteins, including HeV G, are believed to form tetramers (dimer of dimers) at the cell surface [16], [41]–[43], likely held together by disulfide bonds in the stalk region, as well as weaker multi-region dimeric/tetrameric interfaces, including the interface observed here. Notably, the surface area buried in this head-domain dimerization interface is significantly smaller in the henipaviruses (NiV: 714 Å^2^, HeV: 768 Å^2^), as well as morbilliviruses (MeV: 1087 Å^2^ in average), than in other paramyxovirus family members (SV5: 1807 Å^2^, PIV3: 1780 Å^2^) [35]–[39]. It is possible that the reduction in buried area is related to the utilization of cell-surface proteins, rather than sialic acid moieties, as receptor.

### Structure of the HeV-G/ephrin-B2 complex

**Figure 2:**
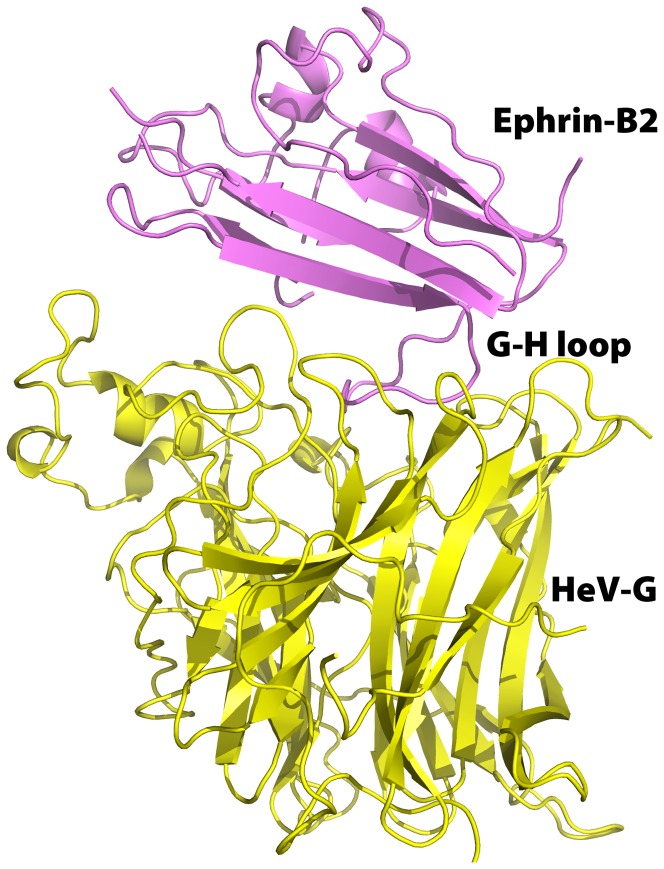
Structure of the HeV-G/ephrin-B2 complex. The HeV-G and ephrin-B2 molecule are colored in yellow and purple, respectively. Ephrin-B2 sits on top face of the HeV-G β-propeller. The G-H loop of ephrin-B2 extends into the central cavity of HeV-G's β-propeller barrel.

**Figure 3:**
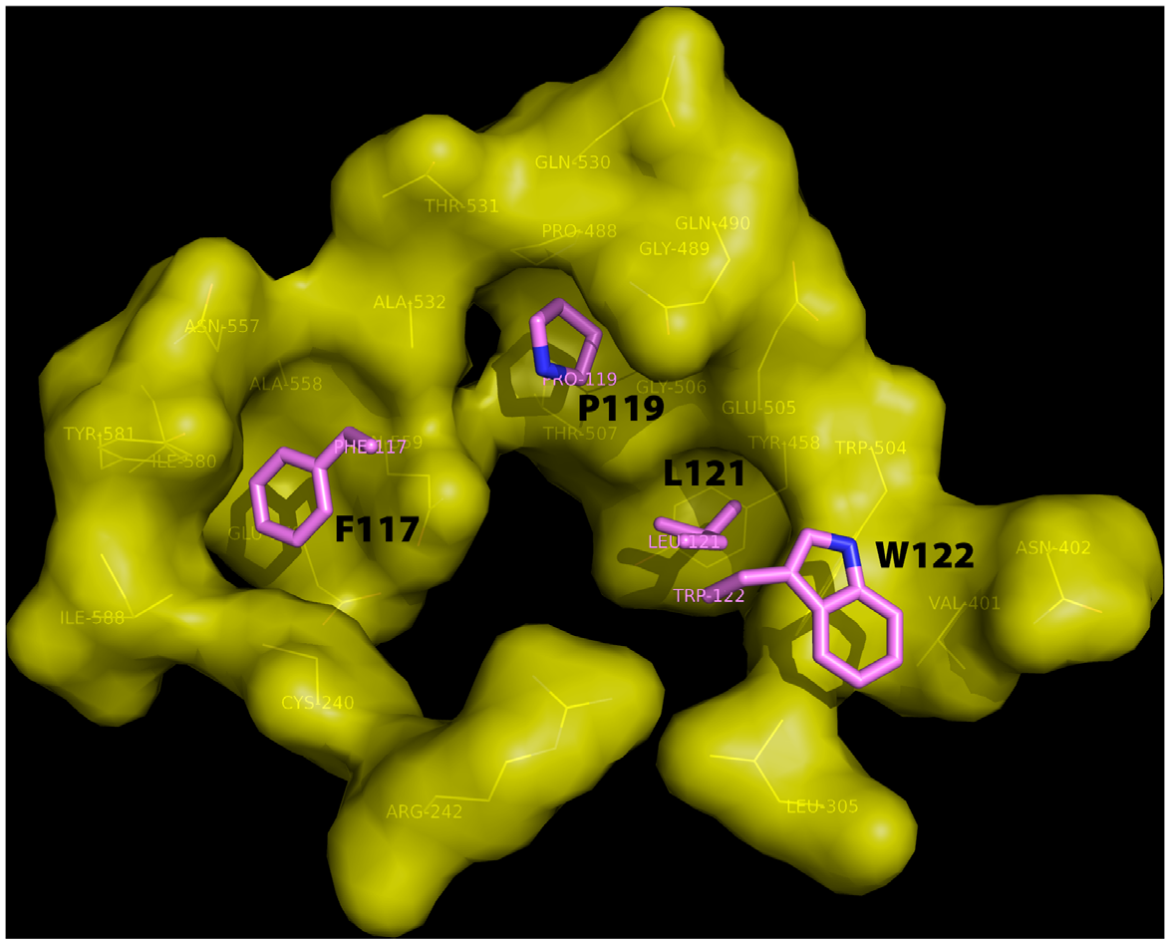
Four hydrophobic residues of the ephrin-B2 G-H loop insert in four hydrophobic HeV-G pockets. The four ephrin residues (F117, P119, L121 and W122) are illustrated as purple sticks. The HeV-G pockets are shown as a yellow surface. The residues defining these pockets are shown as yellow lines and are labeled.

HeV-G and ephrin-B2 form 1∶1 complex both in solution and in the crystals. Four copies of complex were observed in the asymmetric unit of the crystal. The complex buries an interface of 1272 Å^2^, slightly smaller than the 1354 Å^2^ buried in the NiV-G/ephrin-B3 complex and the 1393 Å^2^ - in NiV-G/ephrin-B2 complex. In the complex, ephrin-B2 attaches to the upper face of the HeV-G β-propeller (**Figure 2**) and the ephrin-B2 G-H loop inserts into the HeV-G central cavity. The interface contains a central hydrophobic region and extensive surrounding hydrophilic interactions, including three salt bridges (between residues K103, K113, D105 of ephrin-B2 and residues E501, E533, K388 of HeV-G). In the hydrophobic interface core, four residues (F117, P119, L121 and W122) of the ephrin-B2 G-H loop insert into four hydrophobic pockets in the central cavity of HeV-G (**Figure 3**). The pocket for F117 is formed by C240, N557, A558, Q559, E579, I580, Y581, I588 and R589; the pocket for P119 is defined by P488, G489, Q490, E505, G506, T507, Q530, T531 and A532; the pocket for L121 is formed by Y458, W504, E505 and G506; and the pocket for W122 is defined by L305, V401, N402 and W504.

Ephrin-B2 (S27-D167) used in our structural studies here was expressed in stably-transfected human HEK293 cells. Thus, its glycosylation pattern is more physiologically relevant than in the previously published structures, which used ephrin expressed in either yeast, bacteria or in the presence of glycosylation inhibitor. In addition to the previously observed glycosylation site N36 [32], [44], we now observe another glycosylation site - N139, which is consistent with sequence-based predictions.

Ephrin-B2 undergoes very minor conformational changes upon binding to HeV-G and its structure in the HeV-G/ephrin-B2 complex can be superimposed on the unbound ephrin-B2 structure with the root mean square deviation (RMSD) between equivalent Cα positions of approximately 0.5 Å [44], [45]. The only region on ephrin-B2 that shows some rearrangements upon HeV-G binding is the G-H loop. These conformational changes within this ephrin region are necessary to fit its four hydrophobic residues (F, P, L and W) within the G-H loop into their binding pockets on the HeV-G surface.

### Critical residues in the receptor-binding interface contribute to the distinct receptor-binding properties of NiV-G and HeV-G

**Figure 4:**
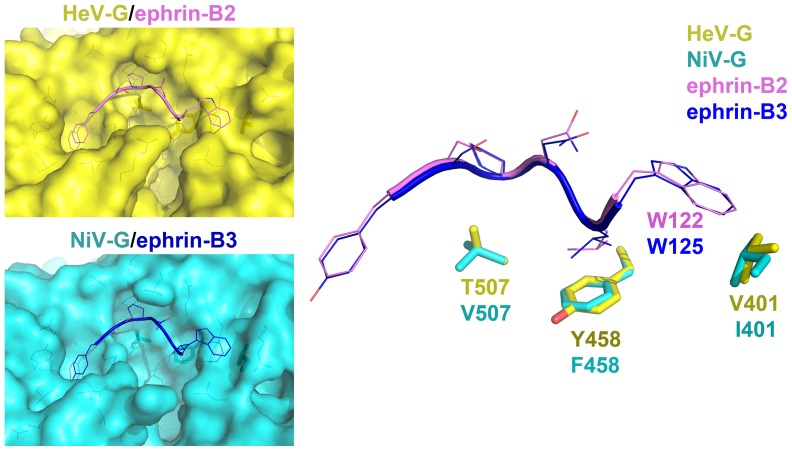
Comparison of the NiV-G/ephrin-B3 and HeV-G/ephrin-B2 interfaces. HeV-G residues are colored in yellow; NiV-G is in cyan. The changes V/T507, F/Y458, I/V401 alter the hydrophobicity of the interface and thus contribute to the different receptor-binding affinities of NiV-G and HeV-G.

It has been suggested that HeV-G has a lower than NiV-G affinity for its receptors and our data here provides a structural basis for this observation. [46] Indeed a comparison of the residues in the receptor-binding region of HeV-G with those of NiV-G reveals that three contact residues of NiV-G (V507, F458 and I401) are replaced in HeV-G with less hydrophobic ones (T507, Y458 and V401) (**Figure 4**). These residues participate in forming the receptor binding pockets for the hydrophobic ephrin (G-H loop) residues P119, L121 and W122 respectively, and the replacements result in a less hydrophobic ephrin-binding channel in HeV-G, and thus a weaker interaction with the receptor.

### Model for HeV-G mediated HeV-F activation upon receptor binding

**Figure 5:**
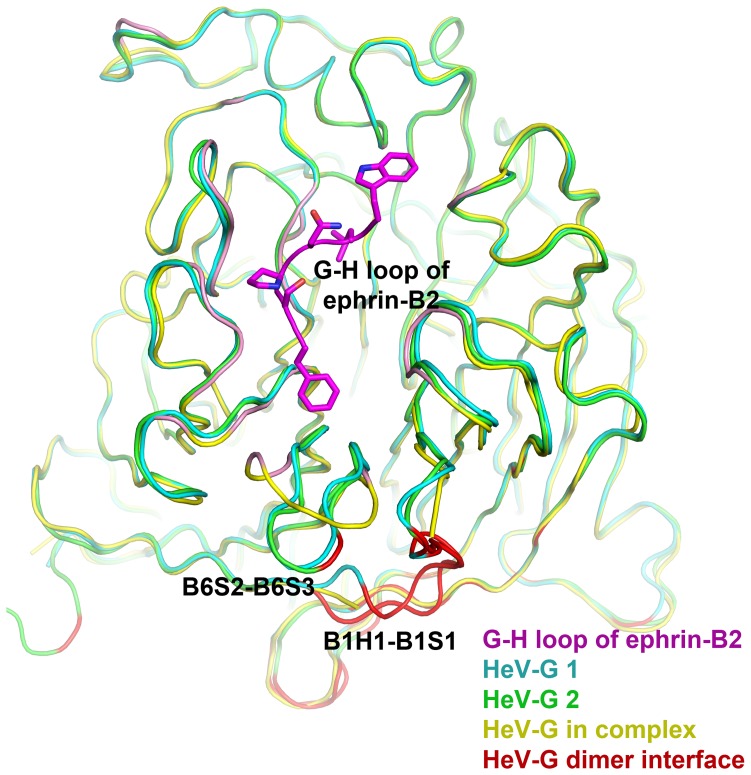
Rearrangement of the receptor binding face of HeV-G upon ephrin-B2 binding. Complexed HeV-G (yellow) is superimposed with unbound HeV-Gs (green and cyan, which represent two different molecules within the same asymmetric unit). The interface between the two HeV-G molecules within the unbound HeV-G homodimer is colored in red. The G-H loop of ephrin-B2 is colored in purple. The ephrin-B2-contacting regions of HeV-G are colored in pink. Receptor binding causes a dramatic conformational change in the B6S2-B6S3 loop region, which then disrupts the HeV-G homodimerization interface observed in the unbound HeV-G.

The structures reported here indicate that the HeV-G head domain dimer is dissociated upon ephrin binding. Interestingly, most of the ephrin-binding residues of HeV-G are in loop regions, with the exception of these forming the F117-binding pocket, which are located on the B6S2 and B6S3 β-strands (**Figure 5**). The positional shift of these residues upon F117 insertion not only locally remodels the F117 pocket, but also causes a significant global conformational change in HeV-G. Specifically, it pushes away the adjacent B1H1-S1 loop region, which as described above is a central part of the HeV-G head domain dimer interface. Consecutively, the ephrin-induced conformational changes in the HeV-G homo-dimerization interface could be the cause of the dissociation of the HeV-G dimers upon receptor binding (**Figure 5**). Thus, the structural data suggests how receptor binding by G could be a trigger for F-activation: Virus in the pre-fusion state possesses F is in its “metastable” (high-energy) state presumably in association with the intact tetrameric HeV-G [24]. Receptor binding causes subtle conformational changes at the HeV-G/ephrin interface which are relayed to the side of the G beta propeller to alter the weak dimeric HeV-G interface and may cause dissociation of the HeV-G tetramers. Following this, HeV-F is triggered and allowed to proceed through a structural transformation into the lower-energy “activated” state which facilitates the membrane fusion process, either by release of inhibition (referred to as the clamp model) or by promotion with the dissociation of HeV-G tetramers (referred to as the provocateur model) [47], [48]. However, we recently extensively characterize several forms of trimeric soluble henipavirus F glycoprotein [49] and reported that it appears that the henipavirus F glycoprotein is expressed in an apparent pre-fusion conformation in the absence of the coexpression of its partner G glycoprotein, strongly suggestion that the ‘clamp model’ as defined may not be accurate. Here, to explore these models further, we analyzed the structures of the four distinct HeV-G/ephrin-B2 complexes within the crystal asymmetric unit and carried out a series of structure-based mutagenesis experiments in HeV-G.

### W122 serves as a “latch” during virus-receptor association

**Figure 6:**
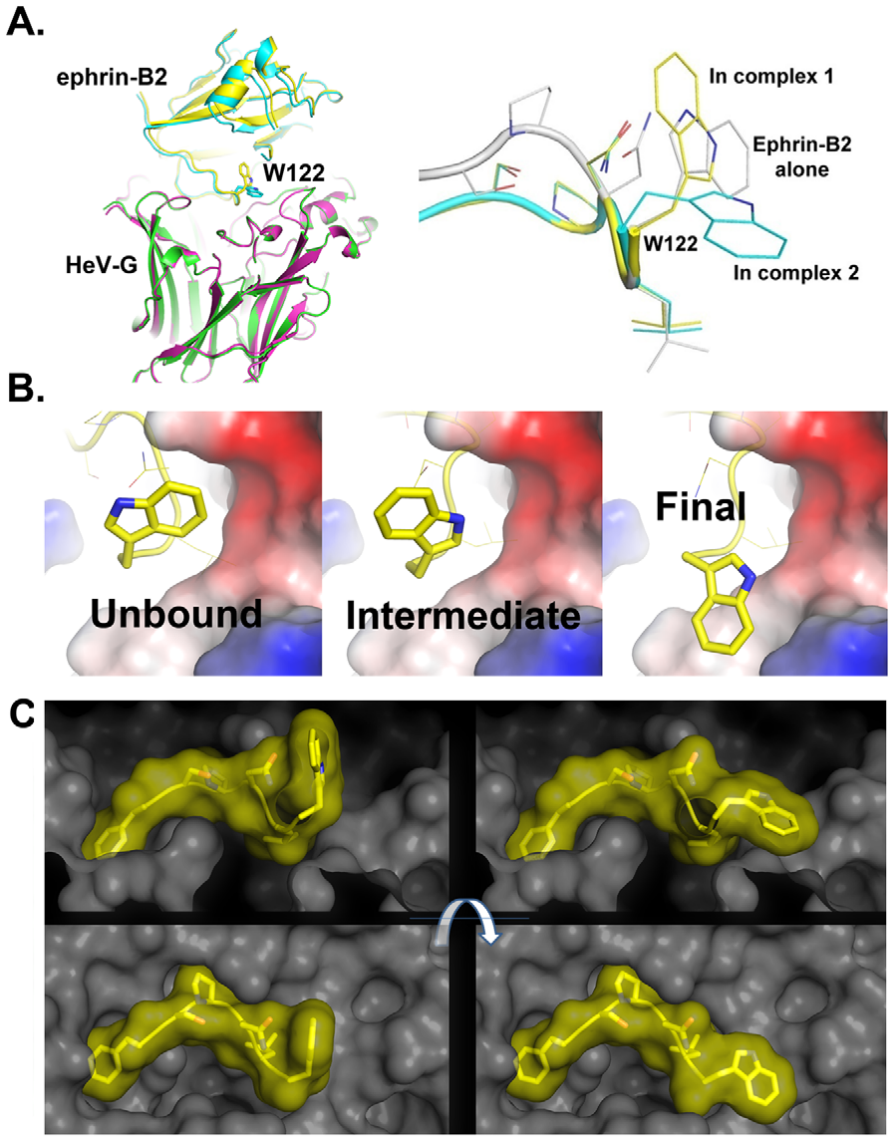
The ephrin-B2 W122 “latch”. Two rotameric W122 (ephrin-B2) conformations in the HeV-G/ephrin-B2 complex, both of which are distinct from the W122 conformation in unbound ephrin-B2. Ephrin-B2 is shown in silver in the unbound state, and in yellow or cyan in the two complexes with HeV-G (A). W122 transforms from the initial unbound conformation to an intermediate conformation upon binding to HeV-G due to steric and electrostatic constrains, then adopts its final conformation via stabilizing van der Waals interactions with HeV-G. W122 is shown as yellow sticks. G is shown as a surface colored according to its electrostatic potential (B). “Latch up” and “latch down” conformations of ephrin-B2-W122 mediate the association and dissociation of the HeV-G/ephrin-B2 complex. Ephrin-B2-W122 is shown in yellow, HeV-G - in grey (C).

Virus-receptor association and dissociation is a dynamic process, which is usually difficult to study using conventional crystallographic methods; however, our crystals provide some very useful snapshots of this process. Specifically, we observed two rotameric forms of W122 in ephrin-B2 among the four HeV-G/ephrin-B2 complexes in the asymmetric unit (**Figure 6A**). In two of the complexes the indole group lies parallel to the HeV-G binding face (“down”). In the other two complexes this group stands perpendicular to the HeV-G binding face (“up”). Interestingly, the “up” rotamer of this tryptophan residue is not observed in any of the related structures (NiV-G/ephrin-B3, NiV-G/ephrin-B2) [32], [50].

The superimposed structures of the G-H loop in the HeV-G/ephrin-B2 complex and in unbound ephrin-B2 are presented on **Figure 6B**, revealing a total of three distinct conformations/rotameric-forms of W122. During receptor binding, W122 is inserted into a groove on the HeV-G surface (**Figure 6B**). The unbound rotamer conformation doesn't fit well because of the groove's shape and localized negative charge. Rotating the side chain to the “up” rotamer conformation results in a snug fit into the binding groove with its amine group facing the electrostatically negative environment of E505, Q490. This conformation is further stabilized by van der Waals interactions with residue L124 of ephrin-B2.

We postulate that the “up” rotamer is an intermediate state and the W122 side chain rotates into the final “down” rotamer conformation, reducing its energy by creating an intricate van der Waals interaction network with the pocket-forming hydrophobic residues in HeV-G. This also helps secure the other three G-binding ephrin hydrophobic residues (F117, P119 and L121) firmly into their pockets, stabilizing the whole central hydrophobic region of the ephrin-G interface. Thus, the structural data supports a “lock, key and latch” model for the association between the G glycoprotein and its receptors with the W122 residue of ephrin-B2 serving as the “latch” (**Figure 6C**). The ephrin “latch” is transiently lifted up as shown on the left panel to facilitate the insertion of the three key hydrophobic residues (F117, P119 and L121) during association of the virus-receptor complex. When the “latch” is pulled down, as shown in the right panel, the aromatic ring edge of W122 is propped against the wall of its binding pocket. This prevents the G-H loop from sliding out and creates an energy barrier for the withdrawal of the other three hydrophobic ephrin residues. We propose that like most protein-protein interactions, the henipavirus receptor attachment event is a dynamic process, during which the ephrin G-H loop inserts and withdraws from the hydrophobic cavity of G. During this process, W122 alternates between rotamers. The occupancies of the “latch-up” and “latch-down” conformations depend on the strength of the hydrophobic interactions within the core of the G-receptor interface. As discussed above, the HeV-G-receptor interface is less hydrophobic and, consequently, of somewhat lower affinity than its NiV-G-receptor counterpart. Accordingly, the dissociation rate is higher, with a higher occupancy of the “latch-up” population.

### Structure-based mutagenesis reveals that the stability of the HeV-G ephrin complex is not stringently correlated with viral entry efficiency

To explore the functional importance of the key interacting elements observed in the binding interface between HeV-G and the ephrin-B2 and -B3 receptors, a series of structure-based alanine substitutions were made in the full length, wild-type (WT) HeV-G, targeting various residues which make up the pockets that accommodate the ephrin-B2 G-H loop residues F117, P119, L121 and W122, or the ephrin-B3 G-H loop residues Y120, P122, L124 and W125. These HeV-G mutations included V401A, N402A, Q490A, W504A, E505A, G506A, Q559A, Y581A, and I588A. In addition, E501A and E533A mutations were also generated, which targeted the HeV-G-ephrin salt bridges at the periphery of the HeV-G-ephrin interface.

**Figure 7:**
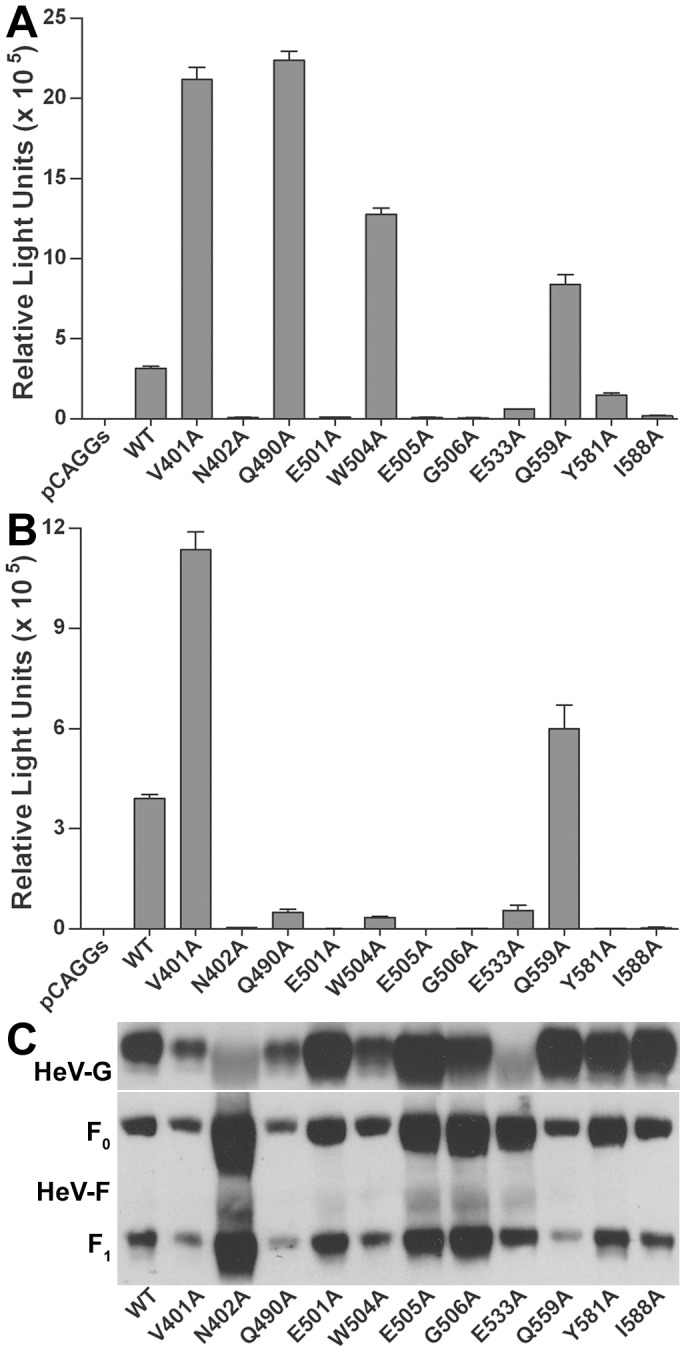
Expression and receptor binding activity of structure-based HeV-G mutations. The various alanine substitution mutants or wild-type (WT) HeV-G were transiently expressed in the absence (A) or presence (B) of HeV-F in HeLa-USU cells. Cell lysates were prepared and equal amounts were co-precipitated with recombinant ephrin-B2/Fc or ephrin-B3/Fc, or directly immunoprecipitated with polyclonal G-specific antibodies (control), followed by protein G Sepharose beads. The precipitated samples were processed and analyzed by 4 to 20% gradient SDS-PAGE and Western blotting with HeV- G-specific antiserum. This experiment was repeated three times and one representative experiment is shown.

We first examined whether the G-glycoprotein mutations effected the HeV-G/ephrin association as measured by co-immunoprecipitation. The series of G glycoprotein mutants were expressed in ephrin receptor negative cells either alone or together with their F glycoprotein partner. Cellular lysates were prepared and a series of precipitation assays were carried out using ephrin-B2, ephrin-B3 or G-specific polyclonal antibodies as a control (**Figure 7**). Each HeV-G glycoprotein mutant was also precipitated from an equivalent amount of lysate with polyclonal antibodies as a control. The results from these experiments were somewhat unexpected as very few of the targeted residues had any measurable effect on the HeV-G/ephrin-B2 association by co-immunoprecipitation, with only W504A showing a significant disruption of ephrin-B2 binding in comparison to wild-type HeV-G (**Figure 7A**). By comparison however, the majority of the HeV-G mutants (Q490A, E501A, W504A, E505A, G506A, and Y581A) revealed a significant disruption in their ability to bind to ephrin-B3 (**Figure 7A**). No differences were observed in the ephrin-B2 or ephrin-B3 binding profiles by any of the HeV-G mutants or wild-type HeV-G when the HeV-F glycoprotein was co-expressed (**Figure 7B**).

### Single substitutions of HeV-G residues within the ephrin-binding interface significantly impact viral entry

**Figure 8:**
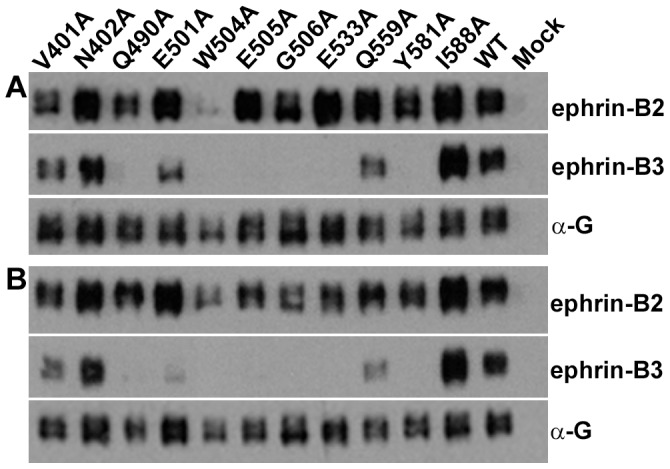
Effect of structure-based HeV-G mutations on viral entry. Luciferase-encoding HIV-1 based pseudovirus stocks were prepared in 293T cells using wild-type (WT) or alanine substitution mutants of HeV-G with the HeV-F by expression plasmid transfection together with pNL4-3-Luc-E^-^R^+^ as described in *Methods*. Each pseudovirus stock preparation was analyzed by p24 quantification and equal amounts of virus particles were used to infect target cells, either Hela-USU cells expressing ephrin-B2 (A) or ephrin-B3 (B), and performed in triplicate. Cells were incubated for 48 hr following infection and processed for luciferase activity quantification using a Centro LB 960 Microplate Luminometer (Berthold Technologies). This experiment was repeated six times and a representative experiment is shown. (C) Incorporation of the HeV F and wild-type and mutant G glycoproteins into pseudovirus particles was assessed by Western blot of lysates prepared from p24 normalized amounts of the same purified virus particles used in Panels A and B. HeV G was detected with a cross-reactive polyclonal mouse antiserum to HeV G and HeV F was detected with a rabbit polyclonal F1 specific antiserum as described in the Methods.

To explore the correlation between HeV-G/ephrin-B2/B3 binding results with the fusion promotion activity of HeV-G, we next examined the impact of the HeV-G mutations on the efficiency of virus entry. Wild-type or the mutant HeV-G constructs were expressed together with HeV-F to generate a series of lentivirus-based, reporter gene encoding pseudovirions as described in the *Materials and Methods* [51]. These HeV glycoprotein-containing pseudovirions were then used to infect either ephrin-B2 or ephrin-B3 expressing target cells. As the structural data suggested, mutations in the interface residues had, in general, a significant effect on virus entry (**Figure 8**). Six of the 11 mutations (N402A, E501A, E505A, G506A, E533A and I588A) completely abrogated virus entry on ephrin-B2 expressing cells (**Figure 8A**), while one mutant (Y581A) a minor inhibitory effect. Interestingly, three of these six substitutions affected electrostatic, rather than hydrophobic HeV-G-ephrin interactions: the K103-E501, and K113-E533 salt bridges, discussed above, and the W122-E505 “latch” interaction which positions the W122 side chain (**Figure 6**). Two mutations (V401A and Q490A) exhibited a notable enhancing effect, which is possibly due, in the case of Q490A, to increase in the hydrophobicity of the binding pockets for the ephrin G-H loop, and, in case of V401A, to facilitate of the transition to the more stable “latch down” conformation of the W122 side chain (**Figure 6**). The W504A and Q559A mutations had a modest yet reproducible enhancing effect on pseudotyped virus entry in comparison to wild-type G (**Figure 8A**).

To further validate these observations, the same series of HeV pseudovirions where used to infect ephrin-B3 expressing cells, and here the effects on virus entry were more profound. Furthermore, in addition to the same G residues showing inhibition upon mutation to alanine, three other amino acid substitutions (Q490A, W504A and Y581A) also completely or significantly abrogated virus entry in ephrin-B3 expressing cells (**Figure 8B**). In addition, 2 of 3 changes (V401A and Q559A) that enhanced the virus entry on ephrin-B2 expressing cells also enhanced virus entry on ephrin-B3 expressing cells. Together, these data reveal an overall similar functional importance of the interface residues within HeV-G for both ephrin-B2 and -B3 receptor usage and also support our W122 latch model. To confirm that impaired function of the HeV-G mutants was not due to a lack of glycoprotein incorporation into the viral pseudotypes, equivalent amounts of each virus pseudotype preparation used in the experiment shown in **Figure 8A and 8B** were lysed and the relative levels of the G and F glycoproteins were measured by SDS-PAGE and Western blot as previously reported [51] (**Figure 8C**). This analysis revealed that most of the HeV-G mutants were incorporated into their respective pseudotyped virus preparations at levels equivalent to or greater than wild-type HeV-G, with exception of the N402A and E533A mutants. The HeV-F glycoprotein was incorporated at levels equivalent to or greater than wild-type HeV F in all pseudotyped particle preparations (**Figure 8C**). Importantly, the entry inhibitory effects of the majority of the HeV-G mutations that either completely abrogated or inhibited virus entry in ephrin-B2 or ephrin-B3 expressing cells (E501A, E505A, G506A, I588A and Y581A), as well as the HeV-G mutants Q490A, W504A which blocked entry on ephrin-B3 expressing cells, were not a result of poor incorporation of the glycoproteins into the pseudovirions.

**Figure 9:**
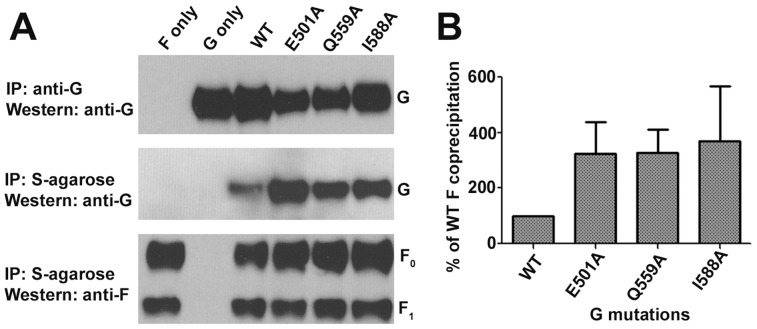
Interaction of HeV-G mutants with HeV-F. (A) HeV-G mutants were co expressed with S tagged HeV-F in HeLa-USU cells. Lysates were immunoprecipitated (IP) with rabbit polyclonal G-specific antiserum to evaluate total G expression (top panel) or S agarose to evaluate total F expression (bottom panel) and co-precipitation of G (middle panel). The precipitated products were analyzed on SDS-PAGE under reducing conditions and then western blot analyzed with F (bottom panel) or G (top and middle panel) specific mouse mAbs. The western blot result of one of three independent experiments is shown in (B). The relative HeV-F binding ability of each HeV-G mutant is shown in comparison to that of WT HeV-G and normalized to total HeV-G expression and the means of three independent experiments are shown. Error bars represent the ranges. The results were calculated using values obtained from digital densitometric measurements of the images.

Thus, most of the mutations, which disrupted HeV entry in ephrin-B2 expressing cells in the context of a pseudotyped virus particle (E501A, E505A, G506A, Y581A, I588A), were not doing so because the mutant G glycoprotein was poorly incorporated into the particles, nor did they have a defect in their ability to bind the ephrin-B2 receptor. The minor difference in the behavior of the W504A substitution in HeV-G, which destabilizes the HeV-G/ephrin-B2 complex, from that of the equivalent mutation in NiV-G, which does not seem to affect the NiV-G/ephrin-B2 binding [52] could be explained by the different receptor binding affinities discussed previously. Notably, two HeV-G mutants (E501A and I588A) were completely abrogated in supporting virus entry into either ephrin-B2 or ephrin-B3 expressing cells, yet they were incorporated into pseudovirus particles along with HeV F at levels equivalent to that of wild-type HeV-G (**Figure 8**) and retained unaltered binding activities to both ephrin-B2 and ephrin-B3 (**Figure 7** **)**. These data indicate that the ephrin receptor binding and its fusion triggering activities on HeV-G can be uncoupled. One possible explanation to account for these observations in the context of the fusion models discussed previously is that these HeV-G mutants no longer associate with its partner F glycoprotein. To examine these mutations in the context of the fusion models, the E501A and I588A HeV-G mutants, as well as the mutant Q559A that appeared to enhance virus entry on ephrin-B2 and ephrin-B3 expressing cells, were tested for their ability to bind HeV-F using a HeV-G/F co-precipitation assay [19] (**Figure 9**). However, the data from this experiment indicated that all three HeV-G mutants possessed no defect in their ability to associate with its partner HeV-F glycoprotein (**Figure 9A**), and even appeared to exhibit somewhat enhanced binding in comparison to WT HeV-G (**Figure 9B**). These findings indicate that HeV-G mutations that are functional in receptor binding and fusion-promotion activity (Q559A) or functional in receptor binding but completely defective in fusion promotion activity (E501A and I588A) can retain a stable HeV-F and G association.

The fact that various mutations at the HeV-G/ephrin interface appear to have little impact on binding while clearly affecting viral entry, suggest that they are likely preventing conformational changes or triggering steps linking ephrin binding with F glycoprotein activation. Indeed the conformational relay from the receptor-binding pocket of the HeV-G protein to its homodimerization interface (**Figure 5**) is initiated by F117 of ephrin-B2 pushing against I588 of HeV-G. Also, the I588A mutation did possess an enhanced association phenotype with HeV-F suggesting that this mutant may also be less able to dissociate from F upon receptor binding or is incapable of inducing F fusion triggering because of an inability to undergo a required ephrin receptor mediated conformational change required for F triggering. However, taken together, our data presented here in conjunction with our findings on henipavirus F [49] suggest that although receptor-induced triggering of the F-mediated fusion process clearly take place, the requirement of G association with its partner F glycoprotein to maintain F in a pre-fusion conformation does not appear apparent, in support of a ‘provocateur model’ of paramyxovirus fusion [48]. Our structures suggest that the I588A mutation would not significantly affect the HeV-G/ephrin binding affinity, but would effectively kill the ephrin-induced global conformational rearrangements in the attachment protein, the data suggest support such a model. N402 and E505 are also involved in the propagation of the ephrin G-H loop-initiated conformational rearrangements to the HeV-G dimerization interface. These data, therefore, indicate that the precise alignment of structural elements at the HeV-G/ephrin interface are directly relayed to affect the productive F fusion triggering. Thus, the alanine mutagenesis experiments support the model for HeV-G mediated HeV-F activation. However, it should be pointed out that our structure based observations were derived using a soluble form of the HeV-G glycoprotein and would not take into account potential long-range effects on the structure of G or its native tetrameric configuration because of the absence of the stalk, transmembrane domain and cytoplasmic tail. Finally, our studies document that targeted mutations, such as I588A, can be designed in the henipavirus attachment proteins, which appear to uncouple host cell/ephrin attachment and viral fusion initiation, providing important tools for additional functional and structural analysis of the precise molecular steps underlying the receptor triggering mechanism of henipavirus entry, and how such mutations in the G attachment glycoprotein accomplish this block.

The structure-based models discussed above also shed light on the dynamic process of the virus-receptor attachment stage, as well as on the mechanism of the G glycoprotein initiated fusion-promotion step that facilitates F activation. The structures provide a clear basis to design further experiments for evaluation of models of henipavirus attachment and entry into host cells and to examine certain key steps in the paramyxovirus entry process in general. Further, the series of structure-based point mutations made in the HeV-G glycoprotein underscore the importance of the individual residues forming the ephrin binding interface, and indicate that both the crystallographically-observed hydrophobic and electrostatic interactions are crucial not only for cell attachment, but also for the subsequent membrane fusion and viral entry events.

In addition, our structure-based mutagenesis experiments reveal three important and unique features of the HeV entry process: First, that single substitutions of interface resides affect more strongly the HeV-G binding and entry to ephrin-B3-expressing cells as compared to ephrin-B2-expressing cells, suggesting that the HeV-G/ephrin-B2 attachment is more robust and resistant to minor structural perturbations; Second, that that the stability of the HeV-G/ephrin association does not strongly correlate with the efficiency of viral entry, suggesting that, particularly for ephrin-B2-expressing cells, viral attachment is not the rate limiting step in the viral entry process; And third, that it is possible to alter the HeV-G/ephrin interface in a way that does not affect the overall stability and/or affinity of the association, but that does affect the efficiency of viral entry, in the context of henipavirus pseudovirions, supporting a model where subtle structural changes at the HeV-G/ephrin interface are relayed at a distance to trigger large conformational rearrangements in the HeV-G-HeV-F complex architecture, initiating membrane fusion.

## Methods

### Construct design and expression of HeV-G and ephrin-B2

Based on the alignment between the HeV G and NiV G glycoproteins, we designed a construct containing residues 171–602, as well as several shorter constructs containing further N-terminal truncations. All HeV-G constructs were subcloned into the pMA152 vector baculovirus expression vector (provided by Alexander Antipenko) with a C-terminal Fc tag and expressed as previously described for NiV-G [50]. The ephrin-B2 construct was designed according to previously published structures [44], [45], [53]. Specifically, the region of residue 27–167 was subcloned into the pCDNA3.1 expression vector with a C-terminal Fc tag to facilitate purification. The plasmid was transfected into an HEK293 (ATCC) cell line using Lipofectamine2000 (Invitrogen). Hygromycin (150 µg/ml) was added for stable–line selection two days after the transfection and well-expressing cell lines were isolated and expanded for large-scale protein production. The medium containing secreted ephrin-B2 was collected and passed through a protein-A column, and the protein was eluted with buffer containing 100 mM glycine PH 3.0 and 150 mM NaCl.

### Protein purification and crystallization

The Fc tag was removed from HeV-G and ephrin-B2 by thrombin cleavage, followed by further purification on a SD200 gel-filtration column (GE Lifesciences) and concentration to 15 mg/ml. Unbound HeV-G was crystallized in vapor diffusion sitting drops against a reservoir containing 20% PEG2000MME and 130 mM (NH4)_2_SO_4_. The HeV-G/ephrin-B2 complex was generated and crystallized as described in [34].

### Data collection and structure determination

Crystals were cryo-protected in mother-liquor with 25% glycerol added. X-ray diffraction data were collected at the Northeastern Collaborative Access Team, Advance Photon Source beamline 24ID-C. The diffraction images were integrated and scaled with DENZO/SCALEPACK [54]. The phases were determined by molecular replacement using Phaser [55] with NiV-G (3D11) and the NiV-G/ephrin-B3 complex (3D12) as search models. The program package PHENIX [56] was used for structure refinement. Manual model building was carried out with the program O [57]. Figures were generated using PyMOL[58].

### HeV-G glycoprotein constructs and mutagenesis

A series of selected alanine substitution point mutations were made in full length HeV-G via site-directed mutagenesis using the Quick-Change II Site-directed Mutagenesis Kit (Stratagene, Cedar Creek, TX). The template for the reactions consisted of a codon optimized full length HeV-G cloned in the pCAGGs expression vector [59]. HeV-G residues mutated to alanine were: V401, N402, Q490, E501, W504, E505, G506, E533, Q559, Y581, and I588. All mutations containing constructs were sequence verified.

### HeV-G and ephrin-B2 and ephrin-B3 co-precipitation

Sub-confluent HeLa-USU cells were transfected for 48 h with the various alanine mutation-containing Gs or wild-type G either alone or in the presence of full length F, using the Fugene-6 transfection reagent (Roche, Indianapolis, IN). Cells were transfected with 3 µg total DNA per T-25 cm^2^ flasks. Cell lysates were prepared using lysis buffer (100 mM Tris-HCl (pH 8.0), 100 mM NaCl, and 1% Triton X-100) and clarified by centrifugation. For co-precipitations of HeV-G with receptors, cell lysates were incubated with 2 µg human ephrin-B3/FC or mouse ephrin-B2/FC (R & D Systems, Minneapolis, MN) followed by precipitation with Protein-G Sepharose (Amersham). As a control and for comparison of expression, equal amounts of lysates were co-immunoprecipitated with 1 µl of HeV-G specific rabbit polyclonal antisera at 4°C for one hr. Samples were washed twice with lysis buffer followed by one wash with 100 mM Tris-HCl (pH 8.0), 100 mM NaCl, 0.1% sodium deoxycholate, and 0.1% SDS (DOC wash buffer). Samples were boiled in sample buffer with 2-mercaptoethanol, analyzed by SDS-PAGE and visualized by Western blotting under reducing conditions with mouse polyclonal HeV-G-specific antisera at 1∶25,000.

### Pseudotyped virus infection assay

Luciferase reporter gene-encoding HIV-1-based pseudovirus stocks were prepared by transfecting 293T cells with plasmids encoding the luciferase virus backbone pNL4-3-Luc-E-R+ [60] along with HeV F and G glycoprotein encoding vectors as previously described [51]. After 4 hr incubation at 37°C, transfected cells were washed extensively with Dulbecco's modified Eagle's medium (DMEM) (Quality Biologicals, Gaithersburg, MD) and incubated for additional 48 hr with DMEM supplemented with 10% cosmic calf serum (CCS) (HyClone, Logan, UT) and 2 mM L-glutamine (DMEM-10) at 37°C in 5% CO2. The resulting pseudovirus containing culture supernatants were clarified by centrifugation for 10 min at 500×g, filtered through a low protein binding 0.45 M filter (Millipore, Bedford, MA) and purified through 25% sucrose in HEPES-NaCl buffer by centrifugation at 36,000×g at 4°C for 2.5 hr. The pellet was resuspended overnight at 4°C in 10% sucrose in HEPES-NaCl buffer and then stored at −80°C until use. Hela-USU cells expressing either the ephrin-B2 or ephrin-B3 receptor (target cells) were infected with a selected amount of the HeV pseudovirions following normalization based on HIV-1 p24 antigen quantitation using an HIV-1 p24 EIA Kit (Beckman-Coulter, Brea, CA). Target cells were seeded into 48-well plates (10^5^ cells/well) and infection experiments were performed in triplicate. After infecting for 2.5-3 hr, cells were washed and incubated for additional 72 hr before lysis with 0.5% TritonX-100 in PBS. A 50 µl aliquot of the resulting lysate was assayed for luciferase activity using luciferase substrate (Promega, Madison, WI). To quantify the incorporation of the HeV-F and -G glycoproteins into pseudotyped HIV-1 particles, equal amounts of sucrose cushion purified virus particles based on p24 content were lysed in buffer containing 100 mM Tris-HCl (pH 8.0), 100 mM NaCl, 2% Triton X-100 and protease inhibitors at 4°C for 30 min. Samples were boiled in SDS-PAGE sample buffer with 2-mercaptoethanol and separated on a 4–12% Bis-Tris gradient gels (Invitrogen), transferred to nitrocellulose, and probed with a cross-reactive polyclonal rabbit antiserum to recombinant HeV soluble G at a concentration of 1∶25,000 or a rabbit polyclonal F1 specific antiserum at a concentration of 1∶25,000, and HRP-conjugated goat anti-rabbit (Pierce Immunochemical, Rockford. IL) as previously described [51].

### HeV-G and HeV-F co-precipitation

S-peptide tagged HeV-F and un-tagged HeV-G encoding plasmids were co-transfected into sub-confluent HeLa-USU cells. HeV-G only and HeV-F only transfections were carried out in parallel as controls. At 48 hrs post transfection, cells were lysed in buffer containing 100 mM Tris-HCl (pH 8.0), 100 mM NaCl, 2% Triton X-100 and protease inhibitors at 4°C for 30 min, clarified by centrifugation, and divided into two equal portions. One half of each cell lysate was used to precipitate the HeV-F glycoprotein and the other half for precipitation of HeV-G. For total HeV-G present for precipitation, 1 µl of rabbit polyclonal anti-G antiserum was added followed by addition of 50 µl of a 20% slurry of Protein G-Sepharose 4B beads. For HeV-F precipitation, 30 µl of a 50% slurry S-protein agarose beads (EMD Biosciences Inc., Madison, WI) was added to a separate and equal amount of lysate. In both cases, the complexed products and beads were washed twice in lysis buffer, and then boiled in sample loading buffer and the precipitated proteins were analyzed by SDS-PAGE and Western blotting under reducing conditions with either F or G specific mouse mAbs. The images of the precipitated F and G glycoproteins were quantified by densitometry using ImageQuantTL Software (GE Healthcare, Piscataway, NJ).

## Supporting Information

Figure S1 **Related to** **Figure 6** **;** **Crystal packing helps to trap the “up” rotamer of W122.** (A) Ephrin-B2 with the “down” W122 conformation contacts the adjacent HeV-G in crystal packing. (B) Ephrin-B2 with the “up” W122 conformation contacts the adjacent HeV-G in crystal packing. (C) Superimposition of the two HeV-G/ephrin-B2 complexes with different conformations of residue W122 (labeled in sticks). The different packing modes in the crystal prevent the “up” and “down” W122 rotamers from switching their conformations.
(TIF)

Table S1 (DOC)
