# Supplementary figures and images for: New Insights into the Hendra Virus Attachment and Entry Process from Structures of the Virus G Glycoprotein and Its Complex with Ephrin-B2
Source: PLoS One. 2012 Nov 5;7(11):e48742. doi: 10.1371/journal.pone.0048742 (PMC3489827; doi:10.1371/journal.pone.0048742)

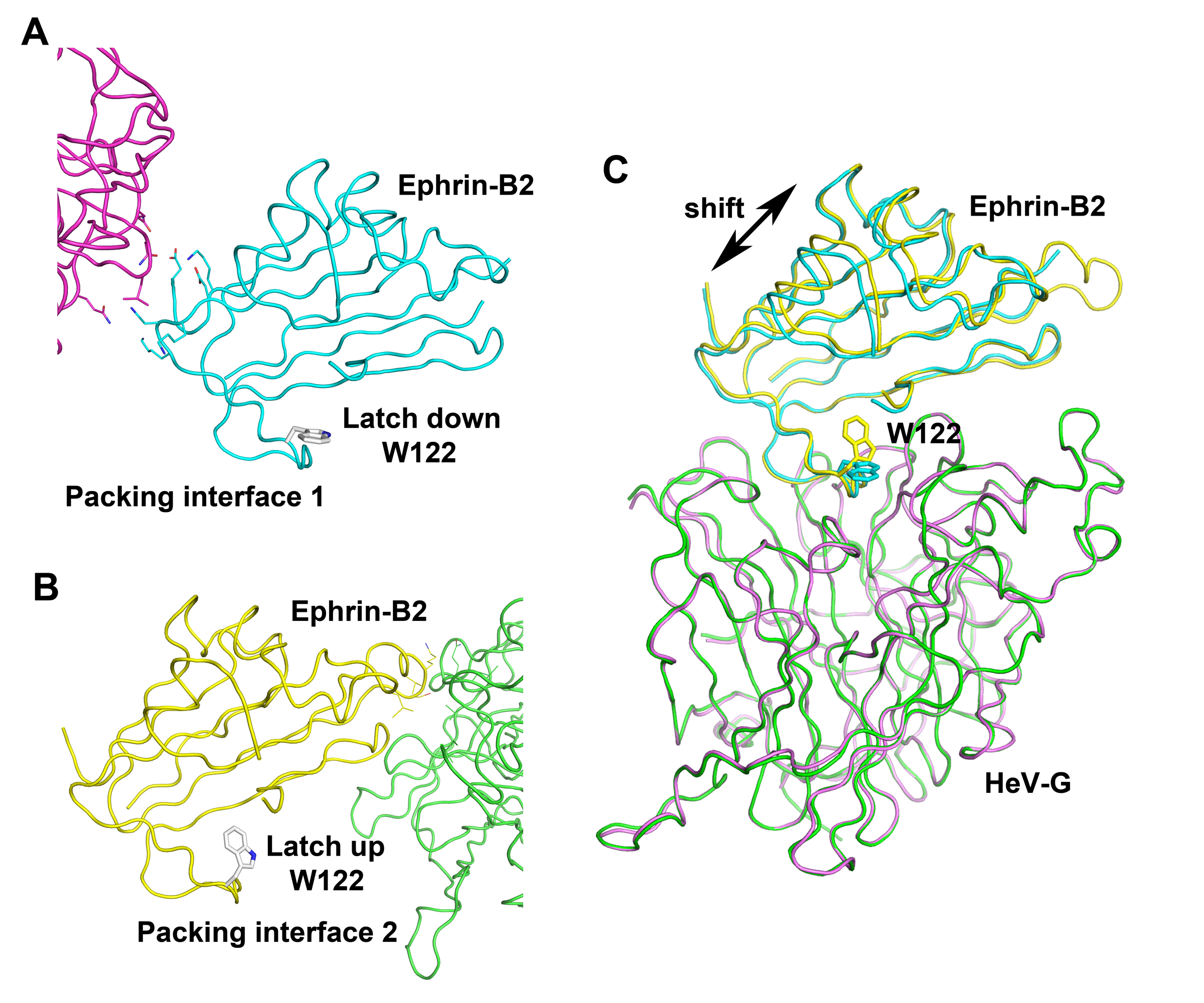

Supplement: Figure S1 — Related to Figure 6 ; Crystal packing helps to trap the “up” rotamer of W122. (A) Ephrin-B2 with the “down” W122 conformation contacts the adjacent HeV-G in crystal packing. (B) Ephrin-B2 with the “up” W122 conformation contacts the adjacent HeV-G in crystal packing. (C) Superimposition of the two HeV-G/ephrin-B2 complexes with different conformations of residue W122 (labeled in sticks). The different packing modes in the crystal prevent the “up” and “down” W122 rotamers from switching their conformations. (TIF) [file pone.0048742.s001.tif]
