# Supplementary material for: New Insights into the Hendra Virus Attachment and Entry Process from Structures of the Virus G Glycoprotein and Its Complex with Ephrin-B2
Source: PLoS One. 2012 Nov 5;7(11):e48742. doi: 10.1371/journal.pone.0048742 (PMC3489827; doi:10.1371/journal.pone.0048742)
Supplement: Table S1 — (DOC) [file pone.0048742.s002.doc]

**Table S1. Summary of crystallographic analysis.**

| **Crystal** | **HeV-G** |
| --- | --- |
| Space group | P21 |
| ASU content | 2 molecules |
| Cell dimensions | a=66.2, b=73.2, c=109.1 Å  α=γ=90°, β=91.85° |
| Resolution (Å) | 2.2 |
| X-ray energy (eV) | 12662eV |
| Completeness (%) | 97.4 (88.6) |
| Redundancy | 3.3 (2.5) |
| *I/ΣI* | 15.5 (3.1) |
| *R*sym (%)* | 7.9 (23.7) |
| *R*free/*R* § | 22.5 / 16.9 |
| r.m.s.d. bond (Å) | 0.004 |
| r.m.s.d. angle (°) | 0.672 |
| Average B factor (Å2) | 30.2 |
| Ramachandran statistics  Most favorable region  Allowed region  Disallowed region | 83.6%  99.7%  0.3% |

Each dataset was collected from a single crystal. Data statistics treats Bijvoët mates independently. The values in parenthesis are for the highest-resolution shells (2.28 - 2.20 Å for HeV-G and 2.80 – 2.70 Å for HeV-G/ephrin-B2).

* *R*_sym_ =∑| *I* -<*I*>|/∑*I*, where *I* is the observed intensity and <*I*> is the statistically weighted absolute intensity of multiple measurements of symmetry related reflections. The values in brackets are for the highest resolution shell.

§ *R* = ∑|*F*_o_ - *k*|*F*_c_||/∑|*F*_o_|, *R* from the working set and *R*_free_ from the test set
